# Neuronal repair after spinal cord injury by in vivo astrocyte reprogramming mediated by the overexpression of NeuroD1 and Neurogenin-2

**DOI:** 10.1186/s40659-024-00534-w

**Published:** 2024-08-12

**Authors:** Zuliyaer Talifu, Chunjia Zhang, Xin Xu, Yunzhu Pan, Han Ke, Zehui Li, Wubo Liu, Huayong Du, Xiaoxin Wang, Feng Gao, Degang Yang, Yingli Jing, Yan Yu, Liangjie Du, Jianjun Li

**Affiliations:** 1 School of Rehabilitation, Capital Medical University; Department of Spinal and Neural Functional Reconstruction, China Rehabilitation Research Center; Chinese Institute of Rehabilitation Science; Center of Neural Injury and Repair, Beijing Institute for Brain Disorders; Beijing Key Laboratory of Neural Injury and Rehabilitation, Beijing, 100068 China; 2 School of Population Medicine and Public Health, Chinese Academy of Medical Sciences & Peking Union Medical College, Beijing, 100730 China; 3 University of Health and Rehabilitation Sciences, Shandong, 266113 China; 4 Department of Rehabilitation Medicine, Peking University Third Hospital, Beijing, 100191 China; 5 Cheeloo College of Medicine, Shandong University, Shandong Province, Jinan, 250100 China

**Keywords:** Spinal cord injury, Astrocytes, Reprogramming, Nerve regeneration, Nerve repair

## Abstract

**Background:**

As a common disabling disease, irreversible neuronal death due to spinal cord injury (SCI) is the root cause of functional impairment; however, the capacity for neuronal regeneration in the developing spinal cord tissue is limited. Therefore, there is an urgent need to investigate how defective neurons can be replenished and functionally integrated by neural regeneration; the reprogramming of intrinsic cells into functional neurons may represent an ideal solution.

**Methods:**

A mouse model of transection SCI was prepared by forceps clamping, and an adeno-associated virus (AAV) carrying the transcription factors NeuroD1 and Neurogenin-2(Ngn2) was injected in situ into the spinal cord to specifically overexpress these transcription factors in astrocytes close to the injury site. 5-bromo-2´-deoxyuridine (BrdU) was subsequently injected intraperitoneally to continuously track cell regeneration, neuroblasts and immature neurons marker expression, neuronal regeneration, and glial scar regeneration. In addition, immunoprotein blotting was used to measure the levels of transforming growth factor-β (TGF-β) pathway-related protein expression. We also evaluated motor function, sensory function, and the integrity of the blood-spinal cord barrier(BSCB).

**Results:**

The in situ overexpression of NeuroD1 and Ngn2 in the spinal cord was achieved by specific AAV vectors. This intervention led to a significant increase in cell regeneration and the proportion of cells with neuroblasts and immature neurons cell properties at the injury site(p < 0.0001). Immunofluorescence staining identified astrocytes with neuroblasts and immature neurons cell properties at the site of injury while neuronal marker-specific staining revealed an increased number of mature astrocytes at the injury site. Behavioral assessments showed that the intervention did not improve The BMS (Basso mouse scale) score (p = 0.0726) and gait (p > 0.05), although the treated mice had more sensory sensitivity and greater voluntary motor ability in open field than the non-intervention mice. We observed significant repair of the BSCB at the center of the injury site (p < 0.0001) and a significant improvement in glial scar proliferation. Electrophysiological assessments revealed a significant improvement in spinal nerve conduction (p < 0.0001) while immunostaining revealed that the levels of TGF-β protein at the site of injury in the intervention group were lower than control group (p = 0.0034); in addition, P70 s6 and PP2A related to the TGF-β pathway showed ascending trend (p = 0.0036, p = 0.0152 respectively).

**Conclusions:**

The in situ overexpression of NeuroD1 and Ngn2 in the spinal cord after spinal cord injury can reprogram astrocytes into neurons and significantly enhance cell regeneration at the injury site. The reprogramming of astrocytes can lead to tissue repair, thus improving the reduced threshold and increasing voluntary movements. This strategy can also improve the integrity of the blood-spinal cord barrier and enhance nerve conduction function. However, the simple reprogramming of astrocytes cannot lead to significant improvements in the striding function of the lower limbs.

**Supplementary Information:**

The online version contains supplementary material available at 10.1186/s40659-024-00534-w.

## Background

The inability of damaged neurons in the adult mammalian central nervous system (CNS) to regenerate and form axons with functional connections is the primary cause of permanent functional deficits once spinal cord injury occurs [1]. The treatment of CNS disorders through stem cell transplantation has been investigated extensively; however, there is still a long way to go before this strategy can be used widely in clinical applications due to ethical issues, low transplantation success rates, immune rejection, tumor formation, and enormous costs [2, 3]. Cellular reprogramming, a process that converts one type of cell to another by manipulating its genetic and epigenetic properties, has been investigated as a potential therapeutic approach for the treatment CNS diseases to regenerate damaged neurons and restore CNS function [4].

As an important approach in the field of regenerative medicine, the original cells become de-differentiated during the reprogramming process into a pluripotent stem cell state and can then be purposefully re-differentiated into specific cell types by various procedures [5]. Several studies have confirmed that glial cells possess full potential to transform into different types of neurons. However, due to the different pathogenic factors and microenvironments of different CNS diseases, the best therapeutic regimens need to be investigated for specific CNS diseases. Thus, different cell therapy strategies need to be developed to treat the pathogenesis of different CNS diseases, the main types of damaged cells, and differences in the physiological environment after damage; all of these factors require the best therapeutic efficacy by combining therapeutic regimens that target multiple targets [4, 6, 7]. Important progress has been made in animal models of brain injury [8], Huntington's disease [9], Parkinson's disease [10], and Alzheimer's disease [11], and multiple transcription factors have been identified that are capable of regulating glial cell reprogramming [5, 12]. In animal models of spinal cord injury, researchers showed that some astrocytes can be converted into neurons and can generate some electrical activity through the regulation of transcription factors such as SRY-box transcription factor 2 (Sox2) and NeuroD1; however, far less research has been undertaken with regards to their overall functional effects [13–15].

In a previous study, Chen et al. [15] found that astrocytes in the spinal cord of rats suffering from spinal cord injury could be reprogrammed into cells with stem cell properties by the direct injection of NeuroD1 recombinant lentivirus into spinal cord tissue. This suggests that it is possible to replenish neurons after injury by applying endogenous methods. When NeuroD1 alone was used for induction, most of the neurons undergoing transformation were glutamatergic neurons. However, when Dlx2 was also administered, the main neurons undergoing transformation were GABAergic neurons; furthermore, the physiological function of the transformed neurons was confirmed by ex vivo electrophysiological techniques [16]. These findings suggest that NeuroD1 regulation may help to initiate astrocyte differentiation, but multi-targeted interventions are needed to be more effective for their directed differentiation to neurons and formation of functional connections. Previously, in vitro experiments revealed that a combination of small molecule drugs was able to reprogram astrocytes into neurons and achieve long-term survival in vitro, and found that up-regulation of NeuroD1 and Ngn2 served as a common intermediate in the different pathways [11]. Ngn2 is a member of the bHLH family along with NeuroD1, and studies have clarified that this family of transcription factors is a key determinant of neuronal cell fate, ensuring that the right number of neurons and glial cells are produced [17]. Ngn2 overexpression in neural stem cells promotes their differentiation into functional neurons; moreover, Ngn2 has been shown to enhance the survival and integration of newborn neurons in the developing brain, and in vivo studies have found that when synergistically overexpressing Nurr1 (nuclear receptor-related 1 protein) and Ngn2 can efficiently convert astrocytes into neurons in the gray matter of the brain [18]; overexpression of Ngn2 alone in the spinal cord induces the emergence of neuron-like cells in the spinal cord that are capable of responding to afferents from dorsal root ganglia [19]. Thus, we hypothesize that the combined overexpression of the transcription factors NeuroD1 and Ngn2 may potentially lead to beneficial effects post-spinal cord injury, significantly enhancing neural regeneration.

In this study, we tested this hypothesis experimentally and found that the modulatory action of key transcription factors can regulate the transformation of astrocytes into functional neurons in cases of spinal cord injury, thereby promoting neurological recovery and reducing glial scar formation.

## Methods

### Experimental animals and grouping

In this study, we used 8-week-old female C57BL/6N mice (Beijing Viton Lever Laboratory Animal Technology Co., Ltd.) which were housed in the Experimental Animal Center of China Rehabilitation Science Institute (SPF-level barrier environment). The experiments were approved by the Animal Experimentation and Laboratory Animal Welfare Committee of Capital Medical University (Reference: AEEI-2021-127).

**Fig. 1:**
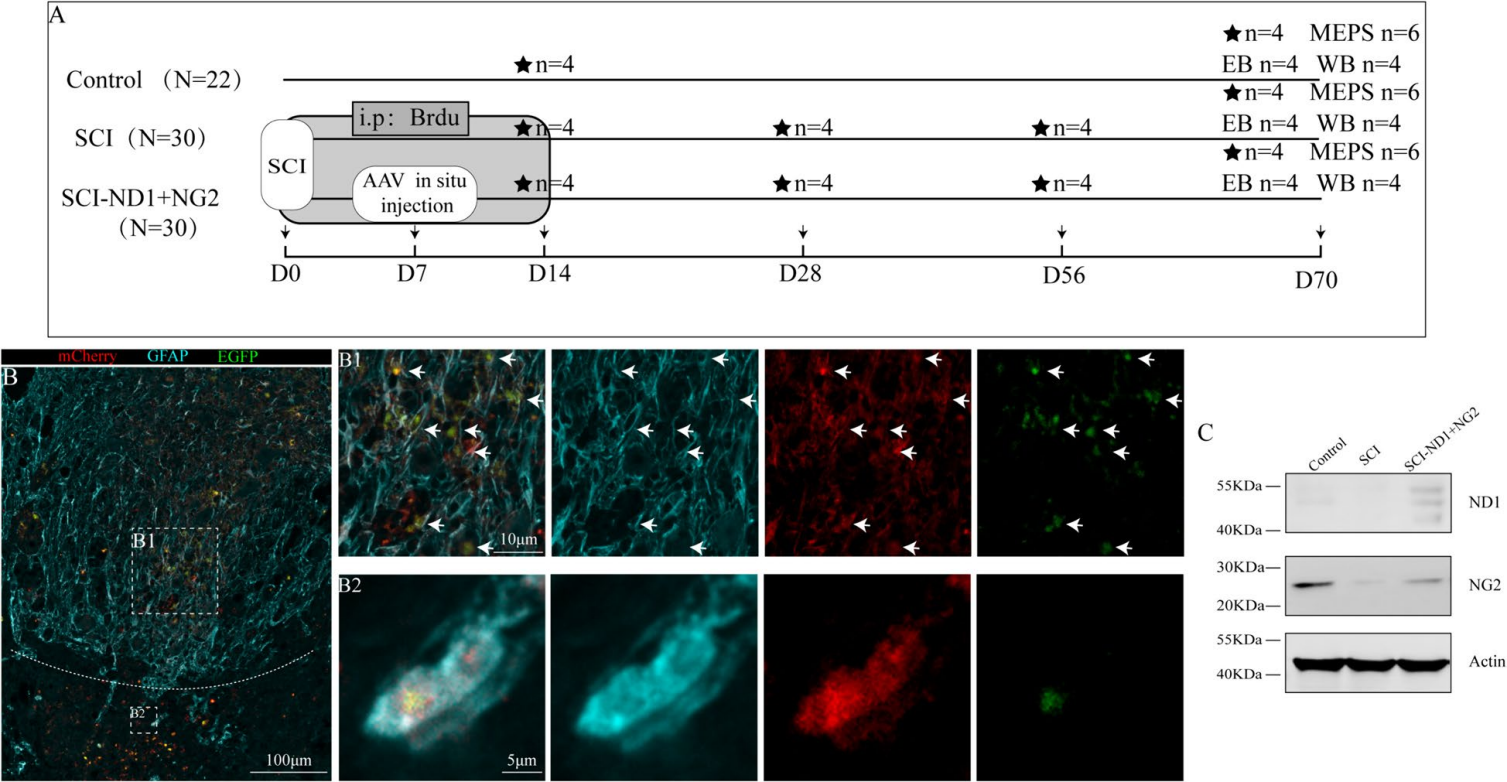
Schematic diagram of the experimental procedure and detection of viral expression in mice. **A** The same batch of mice was randomly divided into three groups, in which the SCI and SCI-ND1 + NG2 groups were modeled for spinal cord injury and underwent continuous BrdU intraperitoneal injection for two weeks to label new neurons. For the SCI-ND1 + NG2 group, AAV spinal cord in situ injection was performed on day 7 after mapping. For each group, four mice were selected for perfusion sampling for immunofluorescence detection on postoperative days 7, 28, 56, and 70, respectively. On postoperative day 70, four animals were selected from each group for WB, six animals from each group for MEPS, and four animals from each group for EB. BrdU: 5-bromo-2´-deoxyuridine, thymidine and nucleotide analogs, i.p: intraperitoneal injection, MEPS: electroencephalographic evoked potentials, EB: Evans Blue assay, WB: protein blotting assay. **B** Representative immunofluorescence results of GFAP (cyan), mCherry (red) and EGFP green at the site of injury; (B1, 2) local magnification schematics; the arrows point to several representative co-localized cells. **C** ND1 and NG2 protein expression levels at the site of spinal cord injury.

The experimental mice were grouped as follows. First, a severe injury model was established by forceps. Mice which showed any form of movement in the lower limbs within 1 day of modeling were excluded while the remaining mice were divided into a target gene NeuroD1(ND1) and Neurogenin-2(NG2) overexpression group (SCI-ND1 + NG2 group) and a SCI non-intervention group (SCI group) by applying a random number method. Healthy mice from the same batch were selected as the normal control group (the Control group). A total of 82 mice were enrolled in the group and the specific experimental protocol is shown in Fig. 1A.

### Spinal cord injury model and AAV injection

The mice were weighed and placed on a holding pad, anesthetized with 1.5% isoflurane, and positioned at the T10 stage of the spinal column. Then, we shaved fur from the area near T10 on the back, sterilized and exposed the muscles spine layer by layer, and used biting forceps to remove the spinous process and vertebral plate of the T9 segment, thus exposing a circular area of approximately 3 mm in diameter with the T10 spinal cord segment as the center. Next, we prepared a model of transverse spinal cord injury using forceps with a tip of 0.5 mm for 5 s (F12013-10, RWD Life science Co., Ltd, Shenzhen, China). Blackening of the transverse spinal cord region and lower limb convulsions were observed after clamping. Then the tissue was provided with adequate hemostasis and sutured layer by layer with a No. 5 absorbable suture. Following suturing, we applied rehydration fluids (saline 0.5 mL) and antibiotics (penicillin 20000U/only) on a daily basis for 3 d to prevent infection. Postoperative assisted voiding care was performed every 12 h until active voiding was resumed. Thereafter, body weight changes were recorded daily. The mice were euthanized with an overdose of pentobarbital sodium overdose if they lost 20% of their body weight after surgery, had a body temperature below 35 °C, or showed self-injurious behavior.

In this study, we selected NeuroD1 and Ngn2 as target regulators and overexpressed their target genes *NeuroD1* and *Ngn2* by administering viral vectors (AAV2/9 serotype) containing the GFAP promoter. Supplementary Figure S1 shows the vector map for HBAAV2/9-GFAP-m-Neurod1-3xflag-EGFP used in NeuroD1 overexpression, while Supplementary Figure S2 shows HBAAV2/9-GFAP-m-Neurog2-3xflag-mcherry used for Ngn2. Seven days after SCI modeling, mice were given 1.5% isoflurane anesthesia and fixed in a stereotaxic instrument. Then, the spinal cord tissues were exposed, and an ultra-micro special positioning syringe pump(QSI, Stoelting, Wood Dale,United States) was used to inject 3 μl of AAV virus solution (1 μl per point) close to the midline at the center of the injury site by a 5 μl microinjector needle (Hamilton, Bonaduz, Switzerland). The coordinates for the injection were 5 mm at the head end and 5 mm at the tail end, at a depth of 1 mm, and a speed of 1 μl/min; the needle was held for 3 min after each point was injected. Successful injection was indicated by the lack of liquid flow-out following removal of the needle. We ensured that each mouse was fully hemostatic and then provided sutures layer by layer. Finally, the mice were placed in a warm environment until they woke up.

### Behavioral assessment

Behavioral assessments were performed as described previously [20]. To prevent subjective interference by the researchers involved, behavior was assessed in a single-blinded manner, which were conducted by a researcher who was not aware of the animal groupings. The mice were placed in the assessment environment in advance to fully adapt before each assessment. Assessments were carried out during the same time period to minimize the influence of diurnal habit differences on behavioral assessments; the main behavioral assessments are described below.

#### The Basso mouse scale (BMS)

The BMS is a widely used scale for assessing motor function in a mouse model of spinal cord injury, and classifies mice into 10 levels of motor function (0 referring to complete paralysis and 9 referring to completely normal motor function). For the BMS, mice were placed in an open field for 15 min prior to assessment; this was followed by acclimatization and 4 min of observation.

#### The open field test

The voluntary motor function of each experimental mouse was evaluated with a TopScanlite device (Clever Sys,Inc. Virginia, United States). Prior to evaluation, mice were placed in the evaluation environment for at least 30 min in advance. During evaluation, mice were placed in a 40 × 40 cm^2^ open field area and their movement trajectories were recorded for 5 min using a high-speed camera. Motor function and emotional state were determined by analyzing the speed of movement, the length of movement trajectories, and the number of times each mouse crossed the central area.

#### The DigitGait test

The motor function of experimental mice was evaluated with a rodent-specific digital footprint analysis system (DigiGaitTM, Mouse Specifics Inc., MA. USA). The speed of the running platform was slowly increased to 5 cm/s and at least four consecutive gait cycles were recorded using a high-speed camera (Basler A602 camera 150 fps).

#### Mechanical pain threshold

The up-down method was used to determine the sensory threshold of experimental mice [21, 22]. This assessment was performed by applying eight different thicknesses (0.02, 0.04, 0.07, 0.16, 1.4, 0.6, 1.0, and 1.4) of Von Frey filaments (Aesthesio, Danmic Global, San Jose, CA). Mice were placed in a plastic cage with a wire mesh bottom that had full access to the foot before evaluation. The mice were acclimated for approximately 30 min until they were allowed to explore the test environment and grooming behavior ceased. A positive response was recorded if the foot retracted, or if the animal licked or jumped up. The force applied at the time of the response was then recorded.

#### Thermal nociception threshold

Plantar thermal nociception was evaluated using an infrared thermal nociception tester (Bioseb, Florida, USA). During evaluation, the mice were placed in a special cage with a glass bottom. Once mice had finished exploratory behavior and grooming, an infrared light source was focused on the soles, and the system would automatically stop timing and display the retraction time threshold when the hind limbs retracted. Three evaluations were performed for each mouse and the mean value was taken at 10 min intervals. To prevent scalding, the maximum test time was 30 s.

### Intraperitoneal injection of BrdU

In this study, BrdU (B5002, sigma) was injected intraperitoneally for two consecutive weeks after SCI to label the newborn cells. First, we prepared a stock solution of 10 mg/mL by fully dissolving the appropriate amount of BrdU with 1X PBS; this was then filtered, de-sterilized, and stored in aliquots at −20 °C to avoid repeated freeze-thawing. BrdU was injected into the mice intraperitoneally at a dose of 10 mg/kg twice daily.

### Blood-spinal cord barrier (BSCB) permeability assay

Mice were injected intraperitoneally with 2% Evans Blue (EB, E2129, sigma) dye (0.4 mL per mouse). After 3 h of deep anesthesia with sodium pentobarbital sodium, the mice were perfused and sampled. Spinal cord tissue at the injury site was harvested, and the fluorescence intensity of spinal EB was observed under a fluorescence microscope [23].

### Electrophysiological evaluation

Motor-evoked potentials (MEPS) in the lower limbs were recorded using an eight-channel electrophysiological recorder (AD Instruments, New South Wales, Australia) in accordance with method described previously with modifications [24–26]. First, mice were anesthetized with 1.5% isoflurane and fixed in a brain stereotaxic apparatus. Then, the head fur was removed and disinfected with an iodophor. The head was then sterilized with an alcohol wipe and the skin of the head was cut using a scalpel. The right cerebral motor cortex was then exposed against the brain atlas using a cranial drill, and two 30-G stimulation electrodes were placed on the cortical tissue in the area of the insertion hole. Recording electrodes were placed on the left gastrocnemius muscle (0.5–1 cm apart) and the reference electrode was placed on the distal part of the ipsilateral lower limb. The stimulation wave width was 1 ms, the current was 7 mA, and the recording electrodes were sampled at 20 kHz. A schematic depicting the assessment is shown in Fig. 10A, B.

### Tissue fixation

Mice were deeply anesthetized with sodium pentobarbital (40 mg/kg, i.p.) and fixed in the supine position on the operating table. Then, the skin and sternum were cut open sequentially to expose the heart. A perfusion pump head was then inserted into the aorta from the apical direction, and the left auricle was quickly cut and perfused, flushed with 0.9% saline, and then fixed with 4% paraformaldehyde solution following the removal of blood. After fixation was complete, we removed the injury site (a 1–2 cm area of the spinal cord tissue at the center of the injury site) and stored in 4% paraformaldehyde solution at 4 °C.

For western blotting (WB), spinal cord tissue was removed following deep anesthesia with sodium pentobarbital. The damaged area was centered to a region of approximately 5 mm in the spinal cord tissue which was snap frozen in liquid nitrogen and then stored at −80 °C to await protein immunoblot assays.

### Immunofluorescence

Fixed tissues were treated with an antigen repair protocol and endogenous peroxidase was removed. Next, we blocked non-specific binding sites and incubated tissues with a range of primary antibodies: NEUN (Abcam, ab177487, 1:3000), MAP2 (Abcam, ab18383, 1:5000), DCX (Abcam, ab18723, 1:5000), GFAP (Abcam, ab7260, 1:500), and BrdU (Abcam, ab6326, 1:200). Next, the tissues were rewarmed and incubated with appropriate secondary antibodies. DAPI was used to stain cell nuclei and tissue autofluorescence was quenched. Finally, the slides were scanned with a TissueFAXS Q + (Tissuegnostics, Vienna, Austria). Data were analyzed with strata Quest (TissueGnostics, version 7.0.1.176) and ImageJ software.

### Western blot

Total protein was extracted from each tissue by weighing an appropriate amount of spinal cord tissue from the site of injury. Protein concentrations were then determined and protein samples were denatured, and separated by SDS-PAGE (G2003, Servicebio). Following electrophoretic separation, proteins were transferred onto membranes. Membranes were then incubated for 30 min with a range of primary antibodies at room temperature: Anti-NeuroD1(Abcam, ab213725), Anti-Neurogenin 2(Abcam, ab109236), Anti-TGF beta 1 (Abcam, ab215715), Anti-SMAD1 + SMAD5 + SMAD9 (Abcam, ab76296), Anti-Smad3 (Abcam, ab40854), Anti-NeuN (Abcam, ab177487), Anti- Smad2 (Abcam, ab33875), p70 S6 kinas (Abmart, T55365), and PP2A alpha + beta (Abmart, T55564). Finally, membranes were incubated with appropriate secondary antibodies and positive signals were detected and imaged with Photoshop (Adobe) and Alapha (Alpha Innotech).

### Statistical analysis

Statistical analysis was performed using SPSS (IBM version 26.0) and R software (version 3.5.3). Data are presented in the form of mean ± standard deviation. For the comparison of two groups of data, the data were first tested for normality followed by the chi-squared test. If the data conformed to a normal distribution, then we applied the two independent samples student-t test; if the data did not conform to the normal distribution, then we used a corrected p-value and performed the Mann–Whitney U test. For the comparison of three groups of data, the data were first tested for normality followed by the chi-squared test. If the data conformed to the normal distribution, then we performed analysis of variance (ANOVA). If the data were not normally distributed, then we applied the Kruskal–Wallis H test.

## Results

### Target gene overexpression promoted the regeneration of damaged cells and the emergence of stem cells

**Fig. 2:**
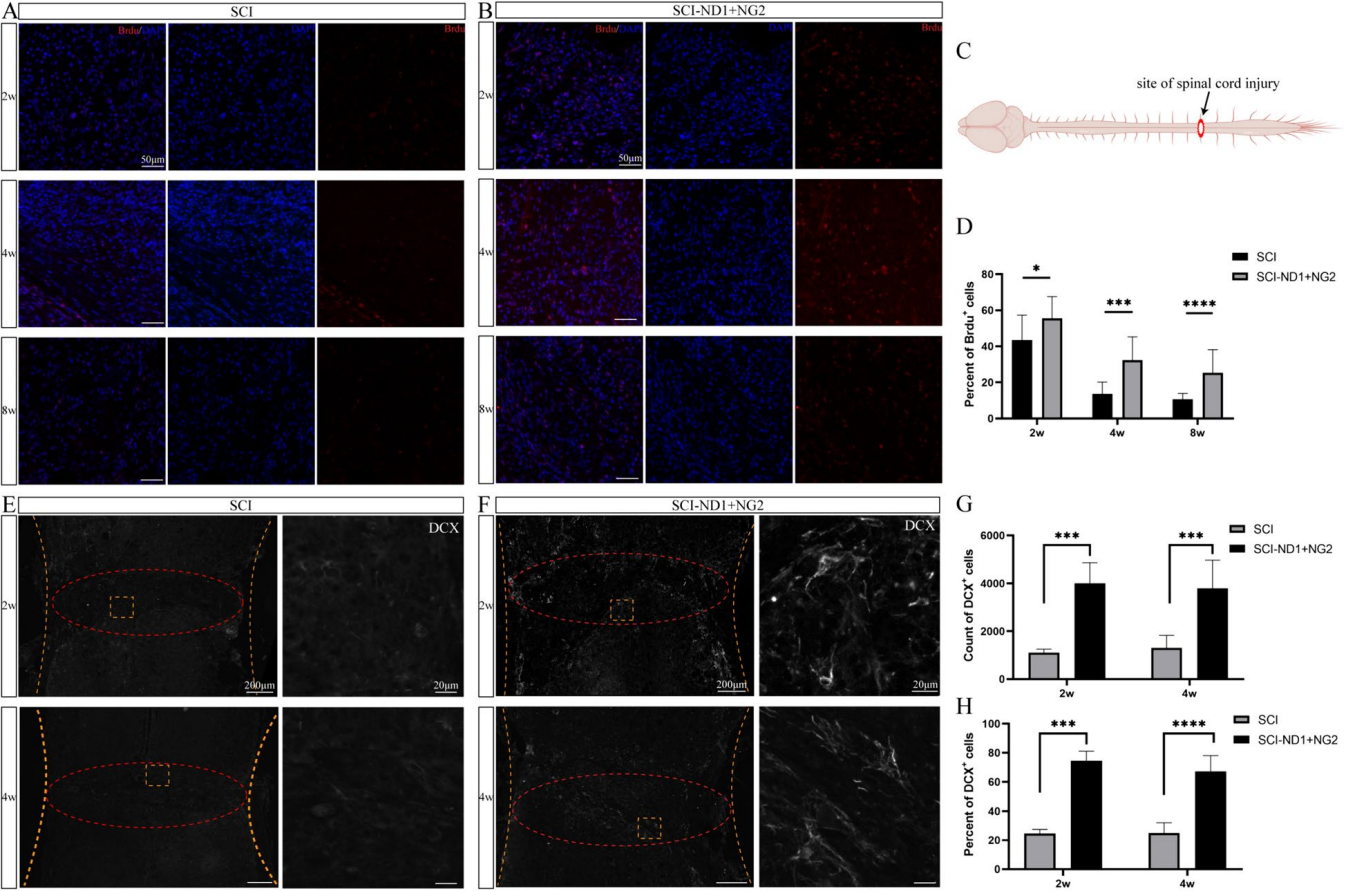
Detection of BrdU and DCX at the center of the injury site. **A** Distribution of BrdU-labeled cells at the site of injury in SCI mice without intervention at 2, 4 and 8 weeks after modeling. **B** Distribution of BrdU-labeled cells at the injury site in SCI mice with target gene overexpression at 2, 4 and 8 weeks after modeling. **C** Schematic diagram of the injury area. **D** The proportion of BrdU neoplastic cells at the site of injury in the two groups of mice at 2, 4 and 8 weeks after modeling. **E** The expression of DCX at the site of injury in the non-intervention SCI mice after 2 and 4 weeks of modelling. **F** The expression of DCX at the site of injury of SCI mice with target gene overexpression after 2 and 4 weeks of modeling. **G** The number of DCX-positive cells at the site of injury in the two groups of mice after 2 and 4 weeks of modeling. **H** The proportion of DCX-positive cells at the site of injury in the two groups of mice after 2 and 4 weeks of modeling. We used the two independent samples t-test and the Mann–Whitney U test. *p < 0.05, **p < 0.01, ***p < 0.001, ****p < 0.0001

Immunofluorescence staining 10 weeks after viral in situ injection revealed a large number of mCherry and EGFP cells co-localized with the astrocyte marker GFAP close to the site of injury (Fig. 1B). New cells close to the site of injury were analyzed by the continuous intraperitoneal injection of BrdU for two weeks after modeling and detected by BrdU-specific antibodies after sampling [24]. The injury area is shown as a schematic diagram in Fig. 2C. We observed a large number of new cells close to the site of injury (Fig. 2A). Overexpression of the target gene significantly increased the proportion of new cells at the injury site; these expression levels decreased after four weeks of SCI, but remained significantly higher in the intervention group than in the SCI group (Fig. 2B, D). Recombinant doublecortin (DCX) can be used as a marker of neuroblasts and immature neurons [27, 28]. In this study, we found that injury induced the appearance of DCX-positive cells at the injury site (Fig. 2D); DCX-positive cells were not detected in other areas of the spinal cord. However, overexpression of the target gene significantly increased the proportion of DCX-positive cells at the injury site (Fig. 2E). Statistical analysis revealed that the total number of DCX-positive cells in the injury area (Fig. 2F) and the proportion of DCX-positive cells (Fig. 2G) were significantly higher than those in the non-intervention group.

### The status of new cells at the site of injury and their characteristics

**Fig. 3:**
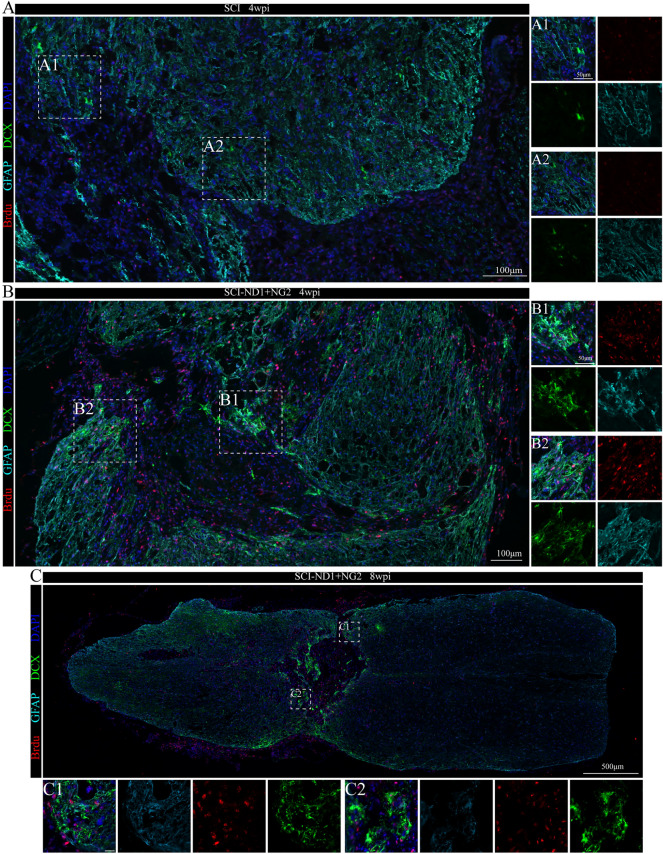
Co-localization of stem cell markers with astrocyte markers and neoblast markers close to the site of injury. **A** Representative immunofluorescence results of GFAP (cyan), BrdU (red), and DCX (green) in the non-intervention SCI mice 4 weeks after modeling; (A1, 2) local magnification schematics, no co-localized cells were observed. **B** Representative immunofluorescence results of GFAP, BrdU and DCX at the site of injury in SCI mice after 4 weeks of modeling; (B1, 2) local magnification schematics, more co-localized cells were observed. **B** Representative immunofluorescence results of GFAP, BrdU and DCX at the site of injury in SCI mice after 8 weeks of modeling; (C1, 2) local magnification diagrams, more co-localized cells were observed

Immunofluorescence staining for the astrocyte-specific marker GFAP, the stem cell marker DCX, and the neoplastic marker BrdU, revealed that there were no co-localized cells at the injury site in the non-intervention group four weeks after spinal cord injury modeling (Fig. 3A). However, there were more co-localized cells in the attachment to the injury area in the two target gene overexpression groups (Fig. 3B). Furthermore, co-localized cells were evident in the area near the center of the spinal cord (Fig. 3B1), in the lateral area (Fig. 3B2). Figure 3B1), and in the lateral region (Fig. 3B2). At 8 weeks post-injury, more co-localized cells were visible in the intervention group (Fig. 3C).

**Fig. 4:**
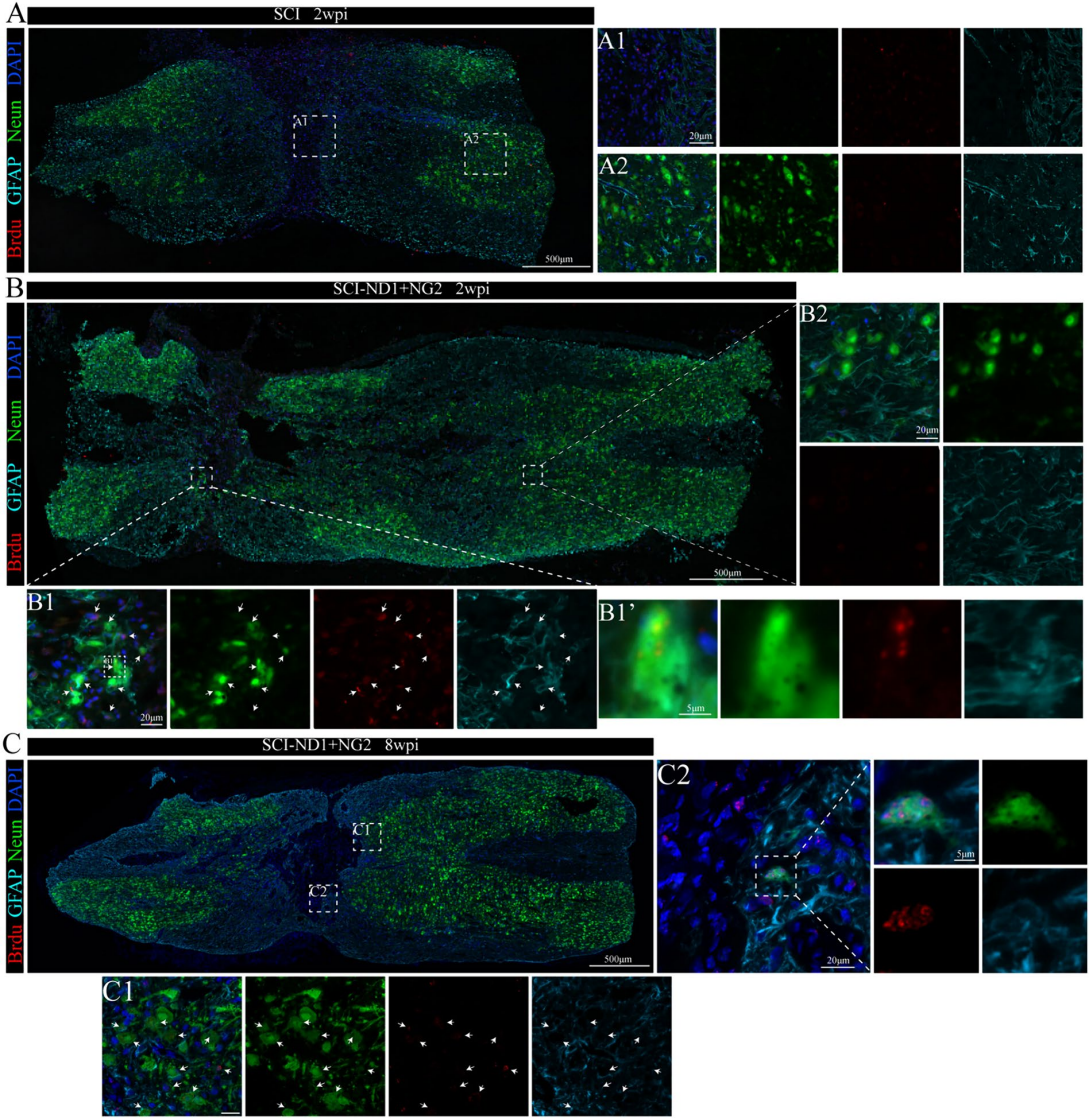
Co-localization of neonatal cell markers with astrocyte markers and neuronal markers close to the injury site. **A** Representative immunofluorescence results of GFAP (cyan), BrdU (red), and Neun (green) at the site of injury in the non-intervention SCI mice 2 weeks after modeling; (A1, 2) local magnification schematics; co-localized cells were not observed. **B** Representative immunofluorescence results of GFAP, BrdU and Neun at the site of injury in target gene overexpressing SCI mice after 2 weeks of modeling; (B1, 2) local magnification diagrams; the arrows point to several representative co-localized cells; (B’) magnification, × 100. **C** Representative immunofluorescence results of GFAP, BrdU and Neun at the site of injury in target gene overexpressing SCI mice after 8 weeks of modeling; (C1, C2) local magnification diagrams; the arrows indicate representative co-localized cells

Two weeks after spinal cord injury, we failed to detect any cells that were co-localized with the neuronal marker NEUN at the center of the injury site (Fig. 4A1) when compared with other regions (Fig. 4A2). Following target gene overexpression, GFAP, Neun, and BrdU co-localized cells were evident at the center of the injury site (Fig. 4B1), but not in the distal part of the injury site (Fig. 4B2). Eight weeks after target gene overexpression, cells showing the co-localization of these three markers were still detectable (Fig. 4C).

**Fig. 5:**
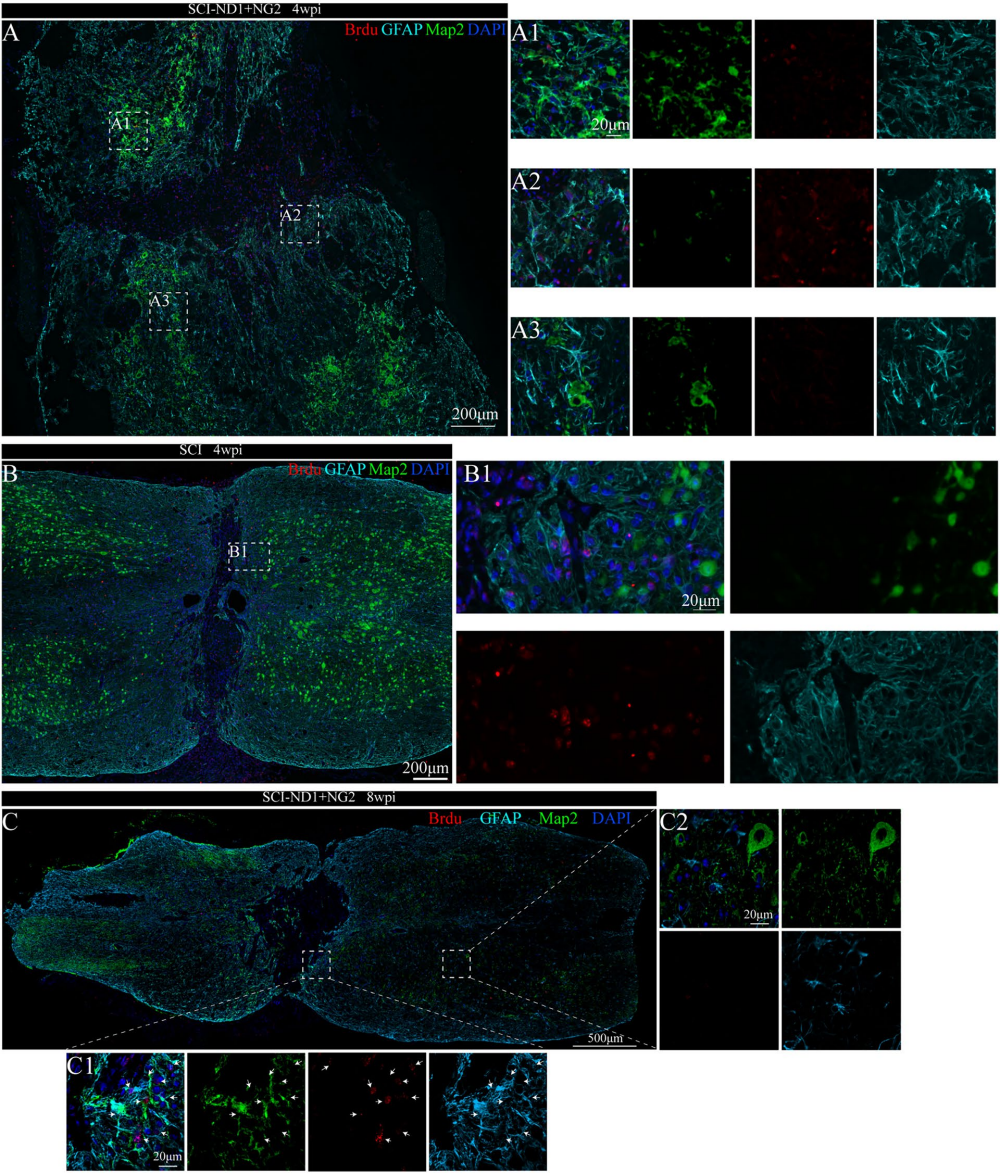
Co-localization relationship of neonatal cell markers with astrocyte markers and mature neuronal markers close to the site of injury. **A** Representative immunofluorescence results of GFAP (cyan), BrdU (red) and MAP2 (green) at the site of injury in SCI-ND1 + NG2 mice after 4 weeks of modeling; (A1, 2, A3) local magnification schematics. **B** Representative immunofluorescence results of GFAP, BrdU and MAP2 at the site of injury in SCI mice after 4 weeks of modeling; B1 local magnification schematics. **C** Representative immunofluorescence results of GFA, BrdU and MAP2 at the site of injury in target gene overexpressing SCI mice after 8 weeks of modeling; (C1, 2) local magnification diagrams; the arrows point to several representative co-localized cells

Cells featuring the co-localization of GFAP, MAP2, and BRDU were also detected 4 weeks after modeling (Fig. 5A), with the highest proportion of co-localization evident at the center of the injury site (Fig. 5A2); this was followed by the area close to the Proximal (Fig. 5A1) and the distal part of the injury site (Fig. 5A3). In the SCI group, although newly generated cells labeled with BrdU were observed near the injury site, no co-localization of GFAP, MAP2, and BrdU was detected (Fig. 5B).

Eight weeks later, more co-localized cells were evident at the center of the injury site in SCI-ND1 + NG2 group (Fig. 5B1), but not in the distal part (Fig. 5B2).

### Motor function

**Fig. 6:**
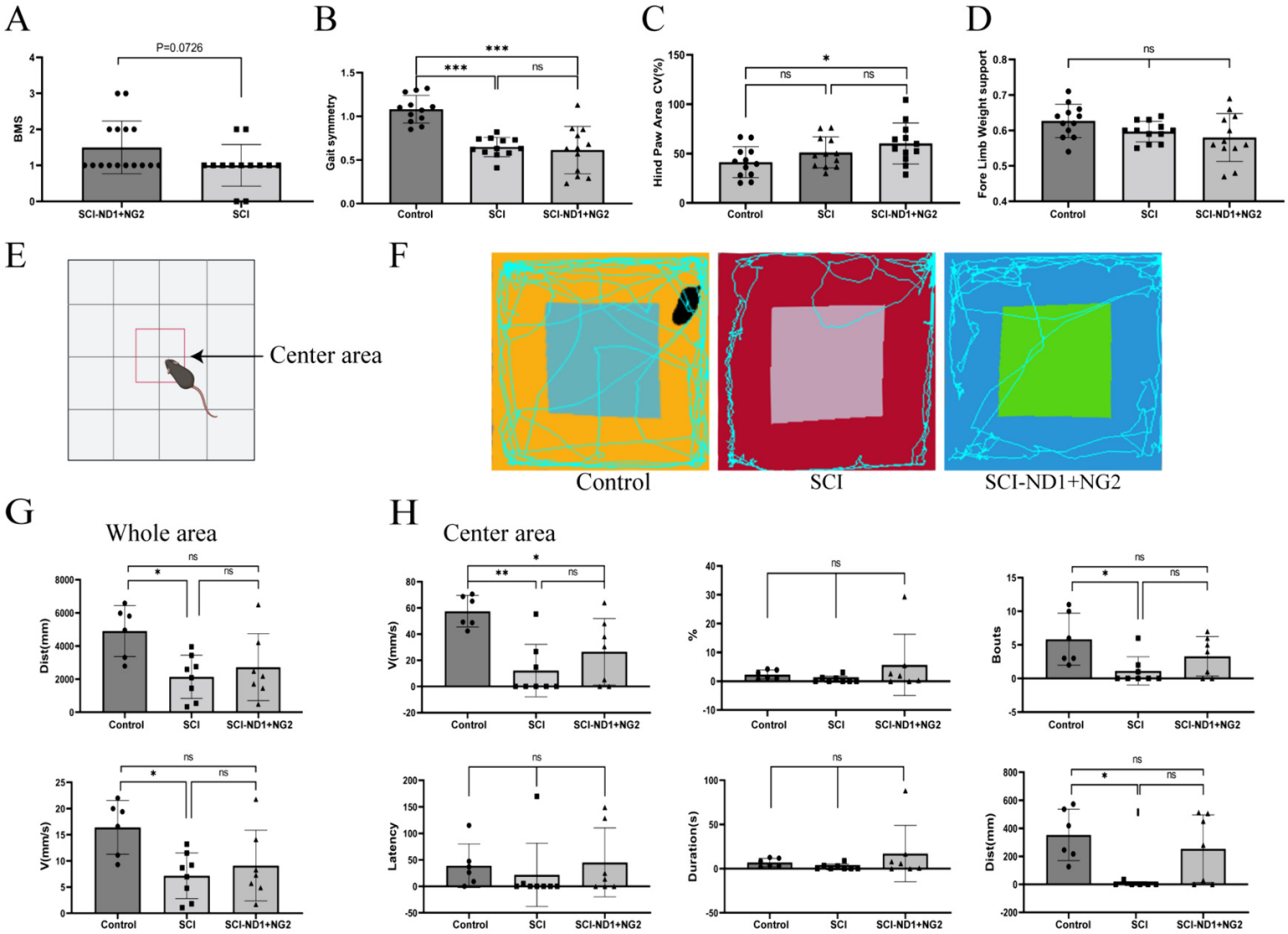
Results of motor function assessment. **A** Results of BMS scores at 10 weeks post-modeling, n = 16 for SCI-ND1 + NG2 group and n = 13 for SCI group, as determined by the Mann–Whitney U test. **B** Results of gait symmetry assessment at 10 weeks post-modeling, n = 12 for each group, as determined by the Kruskal–Wallis H test. **C** One-way ANOVA with n = 12 for each group at 10 weeks post-modeling for the assessment of lower extremity ground area. **D** Upper extremity weight-bearing ratio assessment at 10 weeks post-modeling, n = 12 for each group, as determined by the Kruskal–Wallis H test. **E** Schematic diagram of the open field assessment; the red box indicated by the arrow is the central area of the open field. **F** Representative results of absentee field assessment after 10 weeks of modeling. **G** The length of the trajectory and mean velocity of each group of mice in the whole field after 10 weeks of modeling, as determined by one-way ANOVA/Kruskal–Wallis H test. **H** Statistical analysis of the mean locomotor speed, percentage, locomotor trajectory length, number of times crossing the center, time delay, and duration of each group of mice in the center of the open field after 10 weeks of modeling, as determined by one-way ANOVA/Kruskal–Wallis H test. *p < 0.05, **p < 0.01, ***p < 0.001

The effect of intervention on lower limb motor function was assessed by BMS scores and digit gait analysis. Analysis revealed that 10 weeks after the intervention, the BMS score had not significantly improved in the target gene overexpression group when compared to the non-intervention group (1.5 ± 0.7303 vs. 1.1 ± 0.5774, p = 0.0726) (Fig. 6A). Digitgait analysis revealed that the gait symmetry assessment was 1.0817 ± 0.1584 in the control group and was significantly reduced in the SCI group when compared to the control group (0.6483 ± 0.1104, p < 0.0001). Furthermore, gait symmetry in the target gene overexpression group (0.6125 ± 0.2725) was significantly lower than the control group (p < 0.0001), but there was no significant difference in gait symmetry between the intervention and SCI groups (p = 0.8924) (Fig. 6B). Assessment of the lower limb touchdown area revealed that the coefficient of variation in the intervention group was slightly higher than the control group (60.23 ± 20.73 vs. 51.16 ± 15.69, p = 0.0324), while the difference between the SCI group (42.28 ± 15.71) and both the intervention group (p = 0.3624362) and the control group (p = 0.425) was not significant (Fig. 6C). The upper limb weight-bearing ratio was 0.63 ± 0.05 in the control group, 0.58 ± 0.07 in the SCI group, and 0.60 ± 0.03 in the intervention group, with no statistically significant difference between groups (control/SCI group: p = 0.12, control/intervention group: p = 0.2812, SCI group/intervention group: p = 0.875) (Fig. 6D). We also found that the experimental mice showed a significant reduction in gait symmetry and lower limb reach area after SCI. However, overexpression of target genes failed to significantly enhance the active striding ability and gait of the lower limbs.

In the open field assessment, we found that mice in the intervention group demonstrated greater voluntary locomotion (see Fig. 6F for representative locomotor trajectory recordings). The control group showed the longest and fastest total motor trajectory during the 5 min of assessment; there was a statistically significant difference between the control group and the SCI group although there were no significant differences when compared with the intervention group data (Fig. 6F–G). In addition, the speed of movement in the central region was significantly higher in the control group than in the SCI and intervention groups. There was no significant difference in the number of times the groups traversed the center of the test area and the length of the movement, although the number of times mice from the SCI group traversed the center, and the length of the movement trajectory in the central region, were shorter than those in the control group. However, there was no significant difference between the intervention group and the other two groups. Overall, we observed a trend towards improvement in the mean speed and distance of movement within a fixed period of time following intervention.

### Sensory assessment

**Fig. 7:**
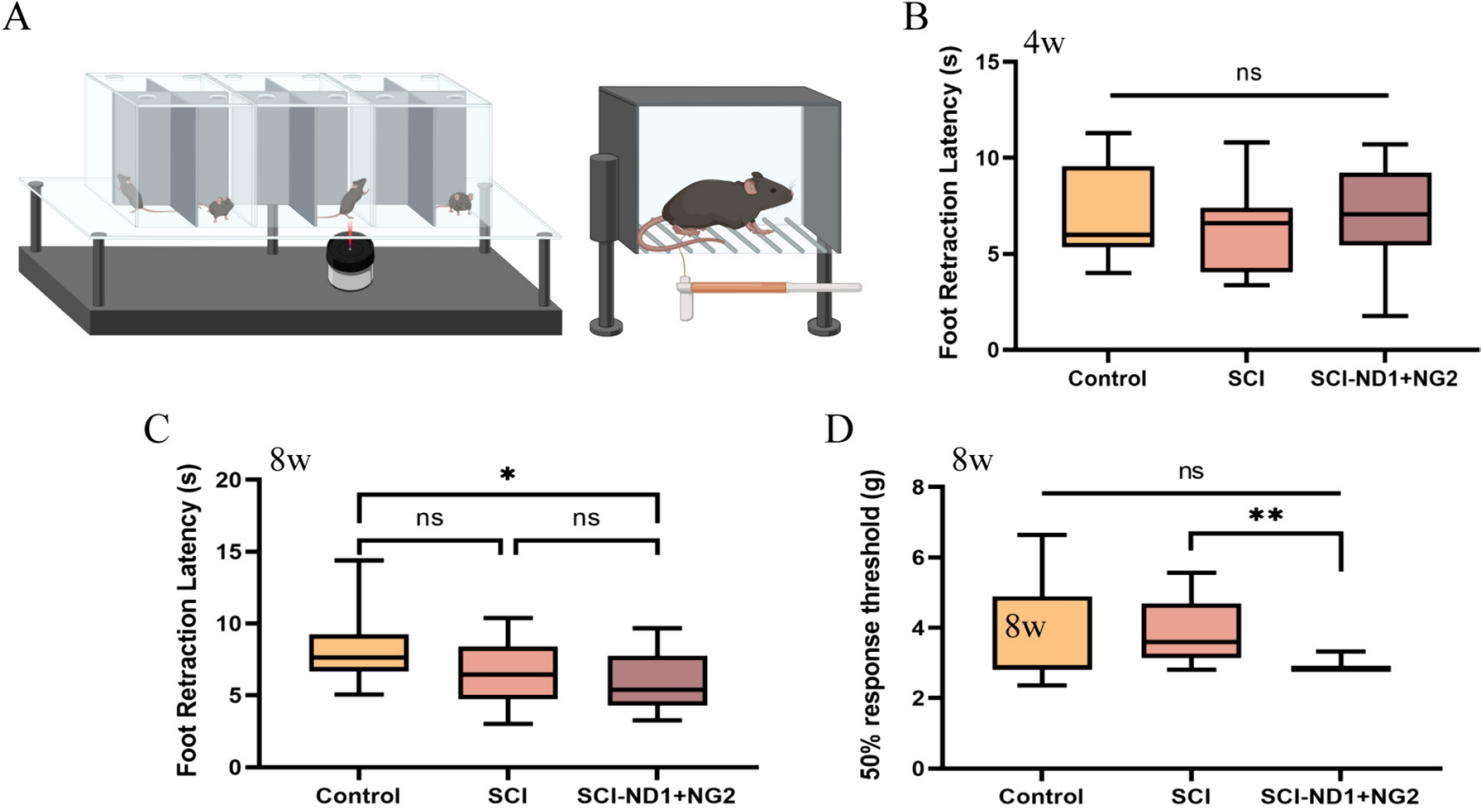
Results of sensory assessment. **A** Schematic diagram of plantar thermal nociceptive assessment (left) and mechanical nociceptive assessment (right) in mice. **B** Results of thermal nociceptive assessment of the lower extremity after 4 weeks of modeling; the vertical axis represents the foot reduction time threshold (s), n = 5 for the control group and n = 6 for the SCI and intervention groups. **C** Results of thermal nociceptive assessment of the lower extremity after 8 weeks of modeling; the vertical axis represents the foot reduction time threshold (s), n = 6 for each group. **D** Threshold of mechanical nociceptive assessment of the lower extremity after 8 weeks of modeling; the vertical axis represents the 50% foot reduction threshold, n = 8 for the control group and n = 12 for the SCI group, as determined by one-way ANOVA/Kruskal–Wallis H test. *p < 0.05, **p < 0.01, ***p < 0.001, and ns represents no statistically significant difference

Sensory assessment revealed no significant differences in terms of thermal nociception between the groups in week 4 (all p > 0.05) (Fig. 7B). By week 8, the duration of thermal nociceptive foot reduction in the intervention group (5.79 ± 1.88 s) was significantly lower than that in the control group (8.09 ± 2.25 s, p = 0.0196); neither group was significantly different from the SCI group (6.69 ± 2.37 s) (Fig. 7C). Plantar mechanical nociception assessment revealed that the 50% foot reduction threshold of the intervention group was 2.87 ± 0.15 g; this was significantly lower than that of the SCI group (3.93 ± 0.89 g, p = 0.0061). The 50% foot reduction threshold in the control group was 3.60 ± 1.58 g; this was not significantly different from the other two groups (Fig. 7D). Collectively, these data showed that the intervention group demonstrated a higher sensitivity to the stimulus and a lower threshold that also induced a positive response to the stimulus.

### The effect of target gene overexpression on the integrity of the BSCB in the spinal cord

**Fig. 8:**
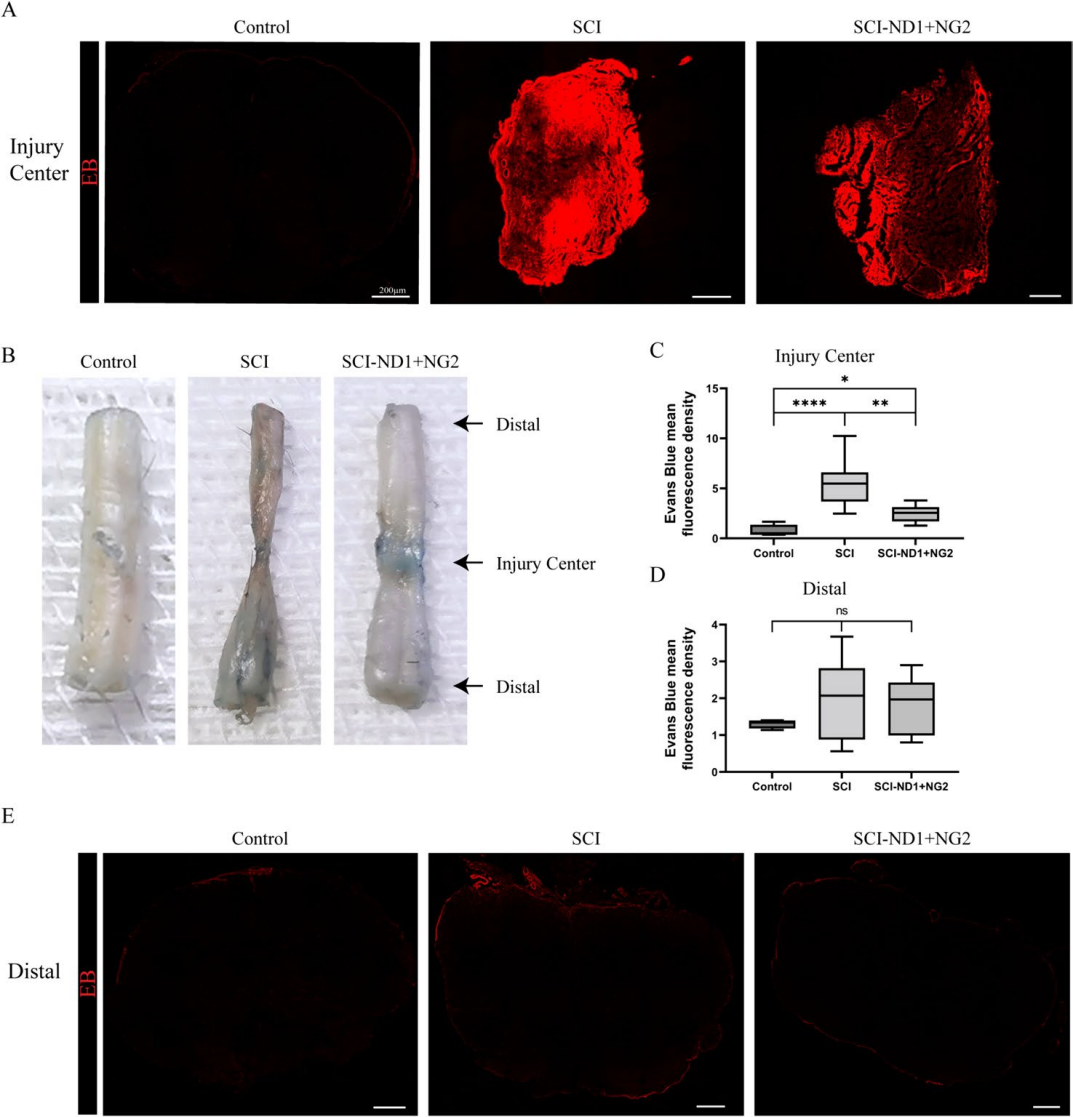
Results of spinal cord barrier integrity assessment after 10 weeks of modeling. **A** Representative results from the EB immunofluorescence assay at the center of the injury site in the spinal cord, with EB immunofluorescence in red. **B** Representative results of histopathological observation of the spinal cord, with EB dye in blue. **C** Quantiative analysis of immunofluorescence intensity in the central region of injury, n = 4 per group, as determined by the Kruskal–Wallis H test. **D** Quantitative analysis of immunofluorescence intensity in the distal region of injury, n = 4 per group, as determined by the Kruskal–Wallis H test. **E** Representative results of EB immunofluorescence detection in the distal region of spinal cord tissue injury; red shows EB immunofluorescence, scale bar = 200 μm. *p < 0.05, **p < 0.01, ***p < 0.001

Following the intraperitoneal injection of EB, we investigated the immunofluorescence intensity of the central region of injury 10 weeks after modeling. We did not detect EB inside the same segment of the spinal cord in the control group (Fig. 8A left), although a strong EB signal was detected inside the entire spinal cord in the SCI group (Fig. 8A middle). The signal inside the spinal cord was weaker in the intervention group than in the SCI group (Fig. 8A right). The contracture of the center of the injured area was significant in the SCI group after 10 weeks of modeling, while the contracture of the center of the injured area was not obvious in the intervention group (Fig. 8B). Quantitative analysis of spinal cord tissue revealed that the immunofluorescence EB intensity was 5.54 ± 2.39 in the SCI group and 2.50 ± 0.84 in the intervention group; this was significantly higher than that (0.74 ± 0.622) in the control group (SCI group/control group: p < 0.0001, intervention group/control group: p = 0.0263). The mean fluorescence EB intensity of the intervention group was also significantly lower than that in the SCI group (p = 0.0016) (Fig. 8C). In contrast, no significant difference was found between the groups in terms of EB immunofluorescence intensity in the distal region of the injury (p > 0.05 for both) (Fig. 8D, E). In summary, this part of the study found that the establishment of the BSCB in the central region of the injury could be promoted by target gene overexpression.

### The effect of target gene overexpression on glial scar formation in regions of spinal cord injury

**Fig. 9:**
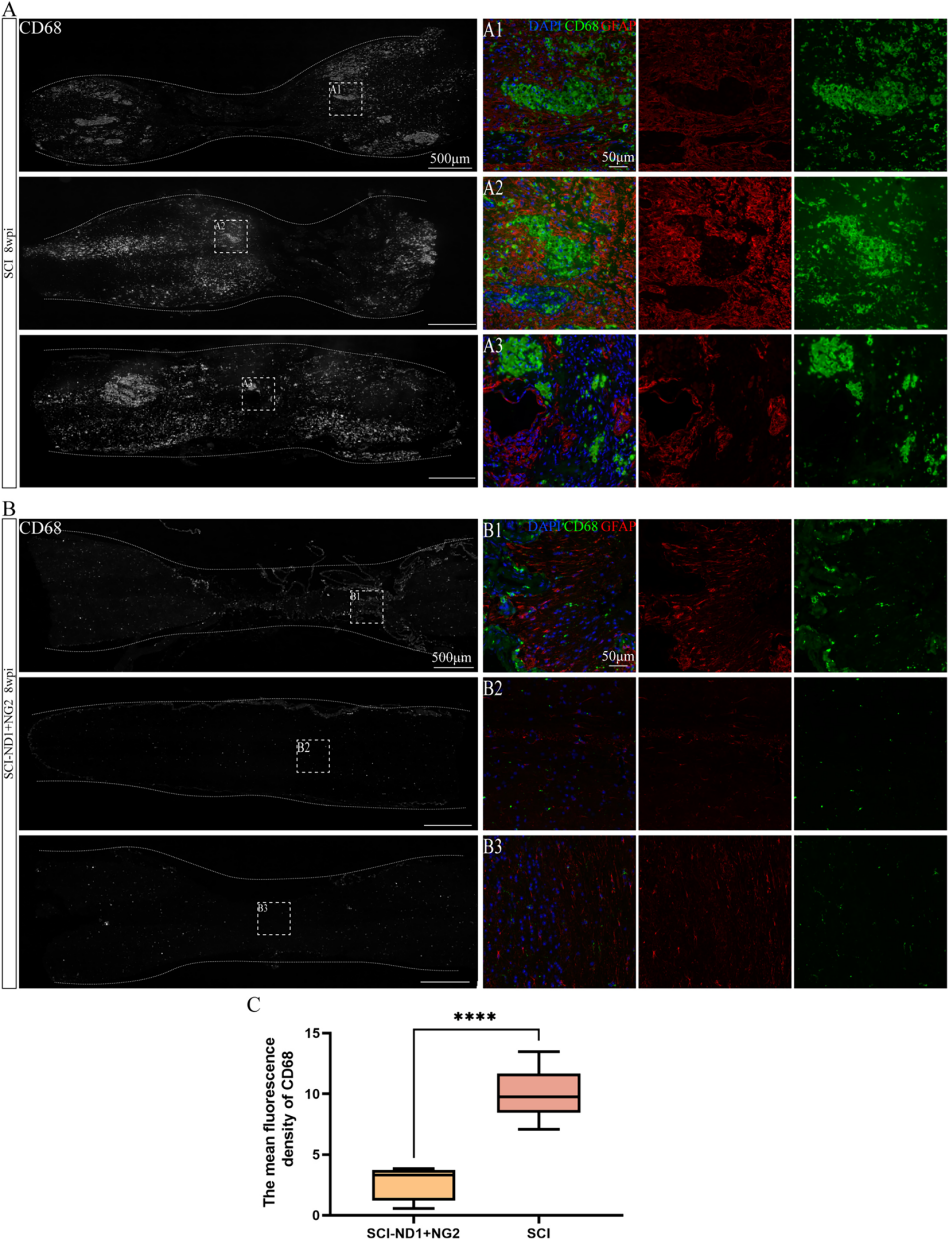
Glial scar formation in spinal cord tissue after 10 weeks of intervention. **A** The right panel shows immunofluorescence staining of spinal cord longitudinal CD68 after SCI, and the local magnification shows the representative immunofluorescence results of GFAP (red), CD68 (green) and DAPI (blue) co-staining in the damaged area of non-intervention mice after SCI. **B** The right panel shows the immunofluorescence staining results of longitudinal CD68 in the spinal cord of target gene overexpressing mice, and the representative immunofluorescence results of co-staining for GFAP, CD68 and DAPI in the damaged area of target gene overexpressing mice after SCI. **C** Quantitative results of CD68 mean immunofluorescence intensity analysis of spinal cord tissues, n = 5 for SCI-ND + NG2 group and n = 8 for SCI group, as determined by the Kruskal–Wallis H test. ****p < 0.0001

Next, we investigated whether the intervention influenced glial scar formation by using the microglia-specific marker CD68 and the astrocyte-specific marker GFAP. After 8 weeks of injury, immunofluorescence analysis showed that the SCI group had more microglia aggregated close to the injury site and produced glial scarring (Fig. 9A), while the intervention group had a lower extent of glial scarring than the SCI group (Fig. 9B). Quantitative analysis revealed that the mean fluorescence density of CD68 in spinal cord tissue was significantly lower in the intervention group than in the SCI group (P < 0.0001, Fig. 9C).

### The effect of target gene overexpression on spinal cord conduction

**Fig. 10:**
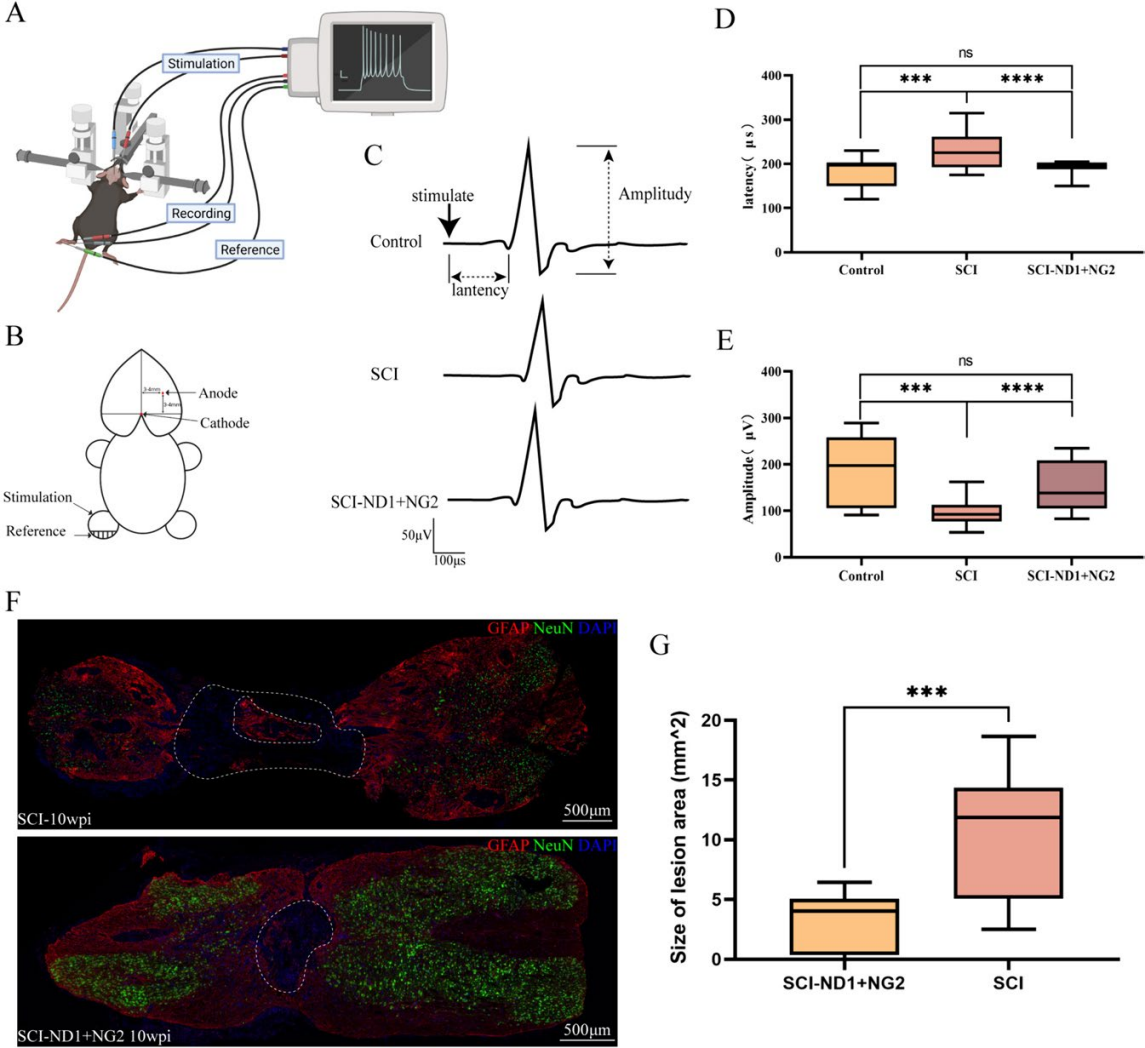
Results of EEG evoked potential assessment and spinal cord defect area assessment in mice after 10 weeks of injury. **A** Representative plots of MEPS assessment results in the control group, SCI non-intervention group and intervention group. **B** The results of MEPS latency statistical analysis, n = 6 for each group; 3–5 waves were taken from each mouse and averaged, as determined by the Kruskal–Wallis H test. **C** Statistical analysis of MEPS amplitude, n = 6 for each group, 3–5 waves were recorded for each mouse and averaged, as determined by the Kruskal–Wallis H test. **D** Representative results of immunofluorescence staining for GFAP (red), NeuN (green), and DAPI (blue) at the site of injury in the spinal cords of mice for the central longitudinal cut of GFAP and NeuN. The upper panel shows the representative results of SCI without intervention, while the lower panel shows representative results from the SCI-ND1 + NG2 group. **E** Results of quantitative analysis at the site of injury in the spinal cord with regards to GFAP area; n = 7 for SCI group and n = 6 for SCI-ND + NG2 group, as determined by the Mann–Whitney U test. *p < 0.05, **p < 0.01, ***p < 0.001, ****p < 0.0001, and ns indicates no significant difference

The ability to transmit information in the spinal cord was analyzed by stimulating the motor cortex and recording electrical signals in the lower limbs. Analysis revealed that mice in the SCI-ND1 + NG2 group exhibited a higher amplitude and shorter latency of EEG evoked potentials than the SCI natural recovery group (see Fig. 10C for representative electrophysiological assessment results). Quantitative analysis revealed that the MEPS wave latency was 190.71 ± 15.75 μs in the control group, 231.17 ± 42.66 μs in the SCI group, and 185.71 ± 25.93 μs in the intervention group, thus suggesting that the latency was significantly longer in the SCI group (p = 0.0005) when compared to the intervention group (p < 0.0001), while there was no significant difference between control group and intervention group (p = 0.9944) (Fig. 10D). The MEPS amplitude was 149.19 ± 52.20 μV in the control group, 98.26 ± 29.07 μs in the SCI group, and 192.87 ± 68.86 in the intervention group. Statistical analysis showed that the MEPS amplitude was significantly lower in the SCI group (p = 0.0001) when compared with the intervention group (p < 0.0001); however, there was no significant difference between the control and intervention groups (p = 0.0896) (Fig. 10E). Electrophysiological assessment suggested that the mice in the intervention group had better spinal cord function in terms of transmitting electrical brain signals after 10 weeks of treatment when compared with the SCI group. Thus, the functional state of the spinal cord had been restored to a better extent in the intervention group.

To determine tissue defects in the spinal cord, we selected mice for GFAP and NeuN staining to observe neuronal cell loss in the injury area. We found that tissue defects were present in both the SCI and target gene overexpression groups at 10 weeks post-modeling (Fig. 10F); however, the area occupied by tissue defects was less extensive in the intervention group than in the SCI group (SCI: 10.41 ± 5.28 mm^2^; SCI-ND1 + NG2: 3.16 ± 2.43 mm^2^, p < 0.001) (Fig. 10G).

### The effects of target gene overexpression on the TGF-β pathway after SCI

After 10 weeks of modeling, Western blot analysis was employed to evaluate the expression levels of proteins associated with the TGF-β pathway at the injury site in the control, spinal cord injury (SCI), and intervention groups. These proteins included TGF-β, Smad1/5/9, Smad2, Smad3, P70 S6, and PP2A.

**Fig. 11:**
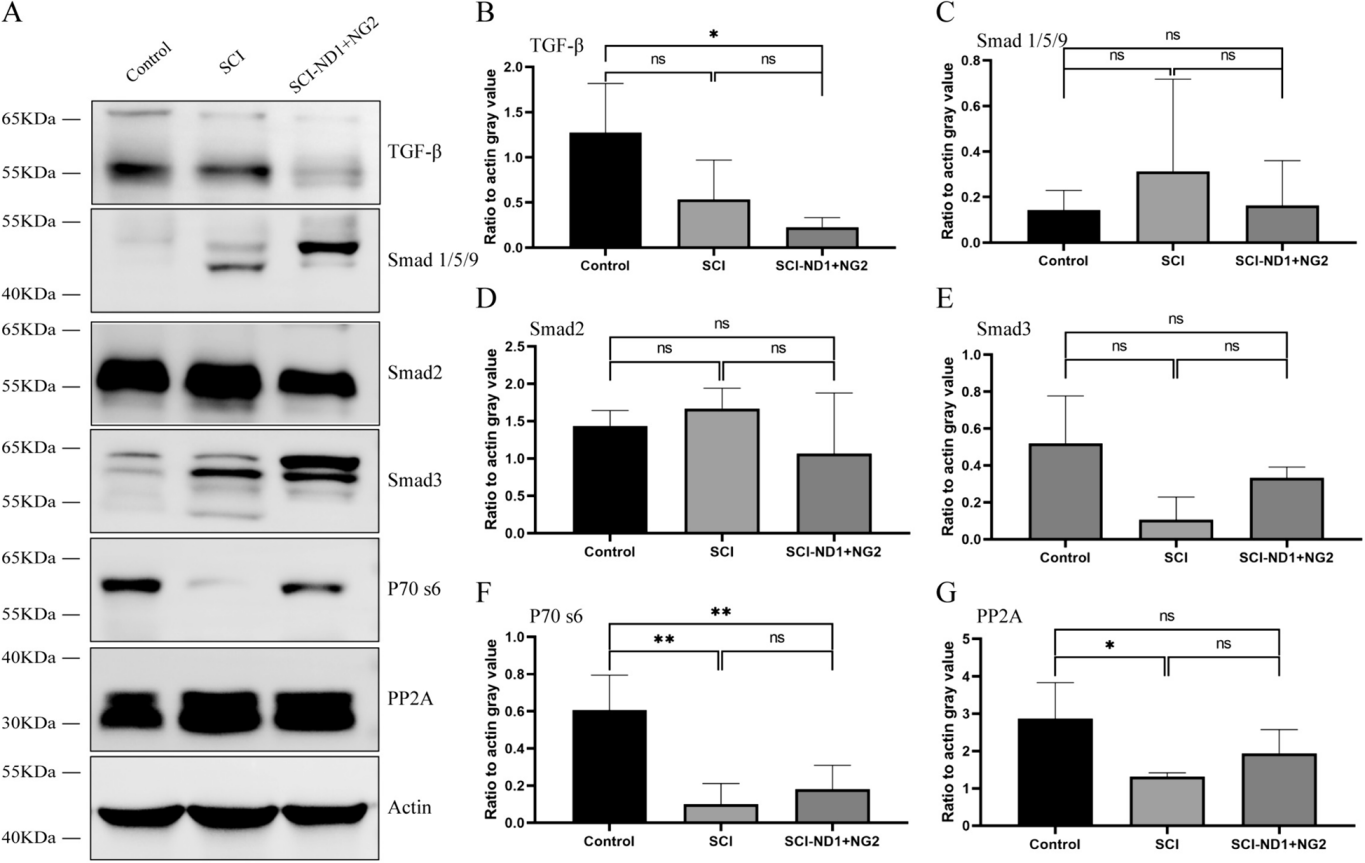
WB assays were used to verify the expression levels of proteins related to the TGF-β pathway. **A** TGF-β, Smad1/5/9/, Smad2, Smad3, P70 S6, and PP2A protein expression levels at the site of spinal cord injury in the control, SCI, and intervention groups, as detected by WB; actin was used as an internal reference control. **B** Quantitative analysis of TGF-β protein, n = 3 per group. **C** Quantitative analysis of Smad1/5/9/ protein, n = 3 per group. **D** Quantitative analysis of Smad2 protein, n = 3 per group. **E** Quantitative analysis of Smad3, n = 3 per group. **F** Quantitative analysis of P70 S6 protein, n = 4 in the control and intervention groups. SCI group n = 3. **G** Quantitative analysis of PP2A protein, n = 3 for each group, as determined by one-way ANOVA/Kruskal–Wallis H test. *p < 0.05, **p < 0.01, p < 0.001, ns represents no statistically significant difference

As depicted in Fig. 11, our findings revealed a significant decrease in the expression level of TGF-β protein in the intervention group compared to the control group (p = 0.0451, Fig. 11B). This reduction suggests a potential downregulation of TGF-β signaling in response to the intervention, which may contribute to the observed improvements in spinal cord repair and functional recovery.

Additionally, we observed significant differences in the expression levels of P70 S6, a downstream effector of the TGF-β pathway, between the SCI and control groups. Specifically, P70 S6 levels were notably decreased in the SCI group compared to the control group (p = 0.0034, Fig. 11F). Furthermore, both P70 S6 and PP2A exhibited significantly lower levels in the SCI group compared to the control group (p = 0.0028 and p = 0.0152, respectively, Fig. 11F–G).

## Discussion

As an integrated aspect of regenerative medicine, cellular reprogramming has not only revolutionized our understanding of the nervous system, but also provided a new way of thinking and a promising strategy for repairing neurological damage [29]. In this study, we successfully overexpressed two transcription factors in astrocytes at the site of injury by overexpressing NeuroD1 and Ngn2. In this study, we achieved overexpression of NeuroD1 and Ngn2 in situ in the spinal cord of a mouse model of spinal cord injury by utilizing AAVs carrying astrocyte-specific promoters. This strategy promoted neural regeneration, and significantly improved the integrity of the BSCB, reduced the area occupied by spinal cord defects, and improved the electrophysiological conduction of the spinal cord. Furthermore, behavioral assessments revealed that mice from the intervention group were more sensitive to sensation and demonstrated enhanced motor function compared to SCI group. By assessing motor function, we found that the intervention failed to significantly improve BMS score and gait, although mice in the intervention group had longer trajectories and faster movements in the open field. Further assessment of sensory function revealed that mice in the intervention group showed more sensitive thermal and mechanical nociception. We hypothesized that this finding was related to reorganization of the spinal cord circuits during neural repair.

The regulation of key transcription factors is an effective step in initiating cellular reprogramming. In this study, we selected NeuroD1 and Neruog2 as regulatory targets, and we hypothesized that the synergistic use of these two transcription factors could activate the gene expression of neurons and suppress gene expression in astrocytes, as reported in previous in vitro and in vivo experiments [8, 16, 30, 31]. When the central nervous system sustains an injury, the site of damage may be in a localized state that is more susceptible to neural regeneration and neural differentiation. A previous study found that stem cell transplantation after spinal cord injury and the concomitant use of inhibitors could induce stem cells to differentiate into neurons and reconstruct spinal cord circuits, thus inducing functional recovery [32]. In the present study, we found that a large number of new cells and a certain proportion of stem cell markers appeared at the center of the injury site in the spinal cord injury and nearby areas. The overexpression of target genes promoted this endogenous regeneration and increased the proportion of cells with stem cell properties, some of which successfully differentiated into cells with mature neuronal properties. However, the endogenous regeneration of cells after spinal cord injury and the mechanisms responsible have yet to be elucidated. Future studies need to investigate these issues and explore how we might improve prognosis by promoting endogenous regeneration.

The BSCB is a complex physiological structure that controls the exchange of substances between the blood and the central nervous system while playing a key role in maintaining homeostasis in the central nervous system [33]. The BSCB plays multiple physiological roles in maintaining the health and function of the central nervous system, and disruption of BSCB functionality can lead to various neurological disorders. Therefore, whether astrocytes, as an important component of the brain-spinal cord-vascular barrier, will have a negative effect on the function of this barrier if its differentiation is artificially regulated needs to be carefully evaluated in future. In our present study, we found that 10 weeks of target gene overexpression exerted influence on the integrity of the BSCB, as was assessed by the intraperitoneal injection of EB. We found that this intervention promoted the repair of the BSCB. We hypothesize that this effect was related to the extensive mobilization of neural regeneration by the two transcription factors, thus promoting repair of the BSCB.

Since different sensations are transmitted by different mechanisms and pathways, we evaluated both thermal nociception and plantar mechanical nociception to fully assess the recovery of circuits related to spinal nociception. We found that the target gene overexpression group showed more sensitive thermal and mechanical nociception, an alteration that has been described as "nociceptive hyperalgesia" in some previous studies [34]. We hypothesize that this phenomenon was related to the reorganization of loops that occur in neural repair, and that the newly generated loops may be more sensitive to stimuli. Findings arising from the open field test may also be related to the difference in sensory recovery between the two groups in that mice in the intervention group would choose to move longer distances because their lower limbs were more stimulated in the open field.

The TGF-β/Smad signaling pathway plays an important role in the regulation of cell growth, differentiation, and development in numerous biological systems, but is also involved in anti-inflammatory, apoptotic, excitotoxic and scar-forming pathological processes [35, 36]. In the current study, we investigated whether our intervention had an effect on the TGF-β pathway and found that the levels of TGF-β were reduced in the intervention group. The expression levels of P70 s6 and PP2A proteins, downstream factors of TGF-β, were increased, we postulate that these changes are intricately linked to alterations in neural regeneration and glial scar formation. Previous research has demonstrated the involvement of the TGF-β pathway in fibrotic scar formation post-injury [37]; remarkably, the use of TGF-β pathway inhibitors has led to a significant reduction in fibrotic scar formation [38]. Inhibiting the TGF-β pathway via SMD2 has proven effective in suppressing fibrotic scar formation, thereby promoting both axonal regeneration and the recovery of motor function [37]. In our investigation, we noted that intervention during the chronic phase following spinal cord injury facilitated the process of spinal cord repair. Analysis of protein content revealed lower levels of TGF-β in the intervention group compared to the naturally recovering group. Furthermore, immunofluorescence staining results indicated a reduced density of glial scars in the intervention group. We speculate that as the neural repair process advances, the levels of TGF-β gradually diminish. Consequently, we hypothesize that direct inhibition of its expression during the chronic phase may also contribute significantly to functional recovery.

The goal of reprogramming reactive glial cells into functional neurons is to reverse glial scars back into the neural tissue and eventually restore the lost neural function. It should be noted that only a small fraction of reactive glial cells could be reprogrammed into functional neurons, with previous studies reporting transfection efficiencies typically around 20% [7]. In the present study, we found that the remaining reactive glial cells still had the ability to proliferate. Several previous reports demonstrated that astrocyte reprogramming can be achieved by applying different strategies [39–41]. However, recent studies have questioned the origin of the newly derived neurons; for example, Tai et al. reported that neonatal neurons could be induced by the exogenous overexpression of the *Sox2* gene; however, the source of these neonatal cells was NG2 glial cells and not astrocytes [42]. Wang et al. [43] reported that neither NeuroD1 overexpression via the AAV vector nor Ptbp1 knockdown could convert astrocytes into neurons; instead, the regenerated neurons were originally present as neurons. Furthermore, leakage of AAV may have led to the reprogrammed neurons reported in previous studies, although the precise mechanisms involved remain unknown. In addition, Wang et al. found that the commonly used viral vector AAV, even when the commonly used astrocyte-specific promoter GFAP was used, differed significantly with regards to the specificity of vector transfection with different serotypes. Four days after in vivo transfection, different serotypes were transfected primarily with astrocytes. However, after 14 days, using commonly used serotypes (8, 9, and PHP.eB), the transfection of astrocytes was significantly lower and fluorescent protein expression began to appear in many preexisting neurons [43]. Similarly, Chen et al. reported that the overexpression of Ptbp1 failed to convert reactive astrocytes into neurons in the brains of a mouse model of PD, as determined by genealogical tracing; it was also noted that the previously reported neurons arising from reprogramming may be pre-existing neurons [44].

Previously, some researchers have expressed concern that the direct transformation of astrocytes may have a potential negative impact on the nervous system. Furthermore, AAV-based gene regulation systems should not be applied for genealogical proof due to problems associated with promoter leakage and non-specificity [45]. Recently, Giehrl Schwab et al. reported the use of an AAV-based integrin split dcas9 activator system (AAV-DCAS) to simultaneously overexpress multiple transcription factors in a mouse model of PD and claimed that this improved AAV vector could avoid many of the existing controversies [46]. Rigorous genealogical tracing has received increasing levels of attention with regards to direct astrocyte reprogramming in vivo, and controversies may persist for some time [47]. Therefore, more specific and safe delivery systems, along with more rigorous experiments, are now needed to resolve these controversies.

## Limitations

(1)Although the use of AAV vector can efficiently transfect astrocytes in spinal cord tissue, the AAV titer used is high and there is a possibility of promoter leakage and transfection of pre-existing neurons, so more attention should be paid to further clarifying the newborn neurons and their origin through the labeling of newborn neurons when determining the results[43, 48]; (2) In order to exclude spontaneous recovery from the study, the spinal cord injury model prepared was a severe injury model, and the rats were percussion-injured, which showed that there was still a large range of tissue loss after 8 weeks/10 weeks. In future studies, more enriching tools, such as the use of bioactive materials to modulate reprogramming, could be combined to achieve a significant functional improvement; (3) Determination of neonatal neuron subtypes: In this study, the determination of neonatal neurons was mainly performed by neuroblasts/immature neurons markers and mature neuron markers, and no clear subtype determination was performed; in future studies, if the induction conditions of motor neurons and the induction conditions of specific types of intermediate neurons can be combined, it may be helpful to achieve spinal cord tissue "precision repair"[29, 49, 50]. (4) Another limitation of our study is the exclusive use of female mouse. While this approach facilitated bladder expression and reduced the risk of complications compared to males, it introduced a potential sex bias. Sex differences in response to spinal cord injury and neurorepair have been documented in the literature, suggesting that findings from female rodents may not fully represent the broader population [51].

## Conclusion

In this study, we investigated the synergistic overexpression of transcription factors NeuroD1 and Ngn2 for the reprogramming of astrocytes after SCI by applying a specific AAV in an animal model. The in situ overexpression of NeuroD1 and Ngn2 in the spinal cord after spinal cord injury successfully achieved the reprogramming of astrocytes into neurons at the injury site and significantly enhanced cell regeneration in the injured area. The reprogramming of astrocytes could influence tissue repair, improve the integrity of the BSCB, and enhance nerve conduction function.

### Supplementary Information

Additional file 1

## Data Availability

All data generated or analysed during this study are included in this published article.

## Abbreviations

SCI: Spinal cord injury
AAV: Adeno-associated virus
Ngn2: Neurogenin-2
BrdU: Bromodeoxyuridine
BrdU: 5-Bromo-2´-deoxyuridine
CNS: Central nerve system
Sox2: SRY-box transcription factor 2
EB: Evans blue
BMS: The basso mouse scale
MEPS: Motor-evoked potentials
WB: Western blotting
NEUN: Neuron-specific nuclear protein
MAP2: Microtubule association protein-2
DCX: Recombinant doublecortin
GFAP: Glial fibrillary acidic protein
DAPI: 4',6-Diamidino-2-phenylindole
ANOVA: Analysis of variance
BSCB: Blood–brain-spinal cord barrier
