# Supplementary material for: Neuronal repair after spinal cord injury by in vivo astrocyte reprogramming mediated by the overexpression of NeuroD1 and Neurogenin-2
Source: Biol Res. 2024 Aug 12;57:53. doi: 10.1186/s40659-024-00534-w (PMC11318173; doi:10.1186/s40659-024-00534-w)
Supplement: Supplementary file 1 — Additional file 1 [file 40659_2024_534_MOESM1_ESM.docx]

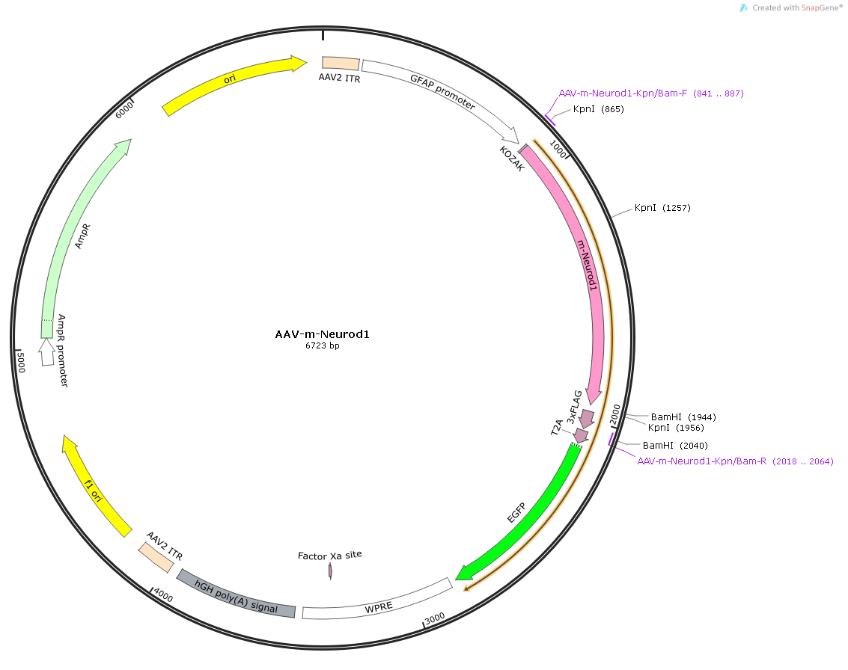


Figure S1. Vector map of the NeuroD1 overexpression viral vector, HBAAV2/9-GFAP-m-Neurod1-3xflag-EGFP.


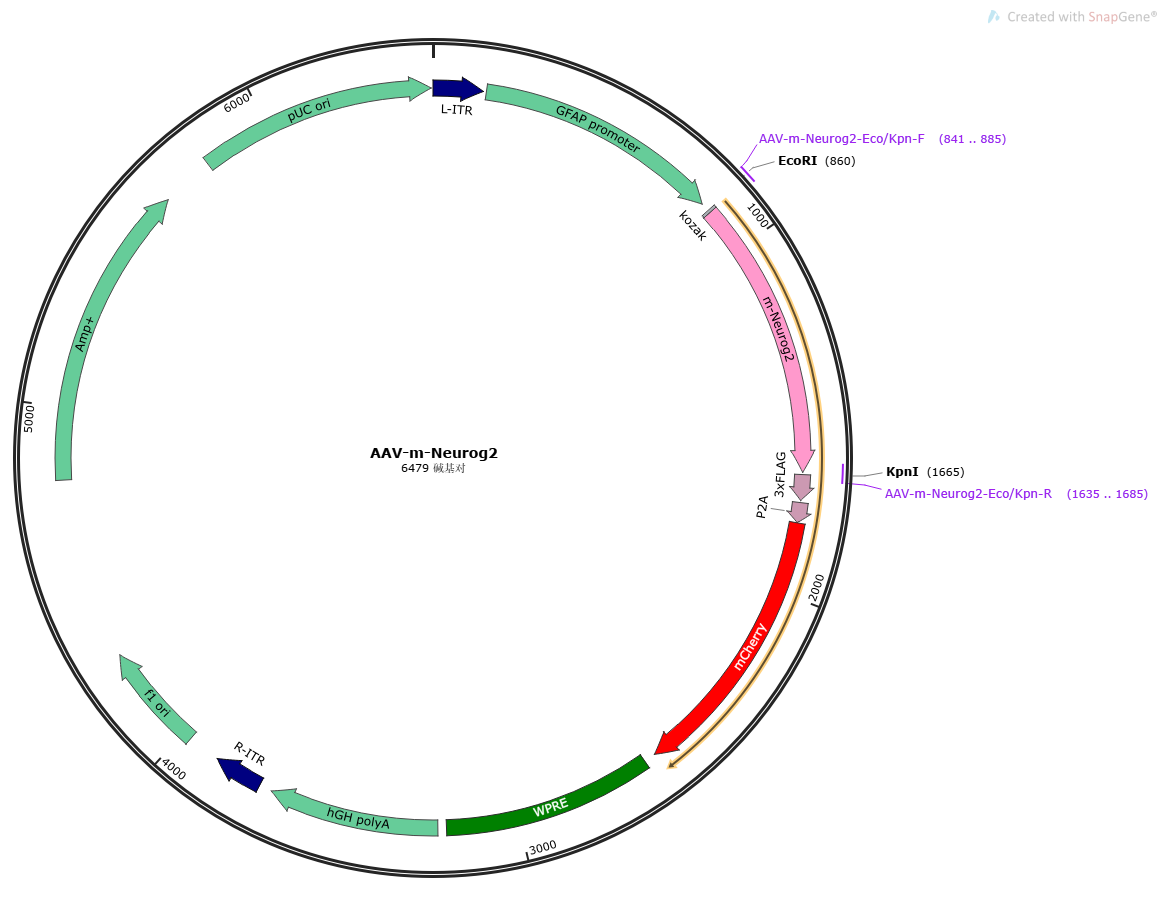


Figure S2. Vector map of the Ngn2 overexpression viral vector, HBAAV2/9-GFAP-m-Neurog2-3xflag-mcherry.
